# Book Reviews

**Published:** 1890-10

**Authors:** 


The seventh edition of DACOSTA'S MEDICAL DIAGNOSIS is now announced by J. B. Lippincott Company as ready. The work has undergone a thorough revision at the hands of its eminent author, and many chapters have been entirely re-written, so as to inculcate all that has been added to our knowledge of disease up to the present time. A number of wood-cuts are included, especially of such micro-organisms as have proved to be of practical significance in diagnosis. All the illustrations are original, and many are from sketches, or based on sketches, taken directly from cases of interest. There is no work more helpful to a young practitioner than this one, which has already been pronounced by eminent critics "the best book on diagnosis extant."

Another valuable book just issued by J. B. Lippincott Company, is Prof. Garretson's TREATISE ON THE DISEASES AND SURGERY OF THE MOUTH, JAWS, FACE, TEETH, and associate parts. Upon the appearance of the first edition many years ago, it assumed the leading place as a text-book, to which its merit, and the distinguished position of its author, entitled it. Much important matter has been added to the new edition, together with numerous illustrations, which greatly increase its value to dentists, surgeons and physicians.

---

CHICAGO POLICLINIC.—The Faculty of this institution have made the following appointments:

Dr. G. Futterer, (late chief assistant to Prof. Rindfleish, of Wurzburg), Drs. F. C. Hotz and E. Fletcher Ingals, Professors of Internal Medicine, Ophthalmology and Laryngology, respectively; also, Drs. Chas. F. Stlllman, P. S. Hayes and J. M. Patton, Associate Professors of Orthopædic Surgery, Electro-Therapeutics and Medicine, respectively.

